# First-Principles Exploration of Hazardous Gas Molecule Adsorption on Pure and Modified Al_60_N_60_ Nanoclusters

**DOI:** 10.3390/nano10112156

**Published:** 2020-10-29

**Authors:** Qi Liang, Xi Nie, Wenzheng Du, Pengju Zhang, Lin Wan, Rajeev Ahuja, Jing Ping, Zhao Qian

**Affiliations:** 1 Key Laboratory of Liquid-Solid Structural Evolution and Processing of Materials (Ministry of Education) & School of Software, Shandong University, Jinan 250061, China; 18536228352@139.com (Q.L.); 17861412028@139.com (X.N.); 201813740@mail.sdu.edu.cn (W.D.); zpj201813800@mail.sdu.edu.cn (P.Z.); wanlin@sdu.edu.cn (L.W.); 2 Condensed Matter Theory, Department of Physics and Astronomy, Ångström Laboratory, Uppsala University, 75120 Uppsala, Sweden; rajeev.ahuja@physics.uu.se; 3 Applied Materials Physics, Department of Materials Science and Engineering, KTH Royal Institute of Technology, 10044 Stockholm, Sweden; 4 College of Traditional Chinese Medicine, Shandong University of Traditional Chinese Medicine, Jinan 250355, China; pingjing@sdutcm.edu.cn

**Keywords:** environment and health, first-principles physics, DFT, electronic structure, hazardous gas

## Abstract

In this work, we use the first-principles method to study in details the characteristics of the adsorption of hazardous NO_2_, NO, CO_2_, CO and SO_2_ gas molecules by pure and heteroatom (Ti, Si, Mn) modified Al_60_N_60_ nanoclusters. It is found that the pure Al_60_N_60_ cluster is not sensitive to CO. When NO_2_, NO, CO_2_, CO and SO_2_ are adsorbed on Al_60_N_60_ cluster’stop.b, edge.a_p_, edge.a_h_, edge.a_p_ andedge.a_h_ sites respectively, the obtained configuration is the most stable for each gas. Ti, Si and Mn atoms prefer to stay on the top sites of Al_60_N_60_ cluster when these heteroatoms are used to modify the pure clusters. The adsorption characteristics of above hazardous gas molecules on these hetero-atom modified nanoclusters are also revealed. It is found that when Ti-Al_60_N_60_ cluster adsorbs CO and SO_2_, the energy gap decreases sharply and the change rate of gap is 62% and 50%, respectively. The Ti-modified Al_60_N_60_ improves the adsorption sensitivity of the cluster to CO and SO_2_. This theoretical work is proposed to predict and understand the basic adsorption characteristics of AlN-based nanoclusters for hazardous gases, which will help and guide researchers to design better nanomaterials for gas adsorption or detection.

## 1. Introduction

In recent years, industrial and fossil-fuel motor exhaust gases and the flue gas from the burning of garbage and straw crops in some areas have come to harm humans and the environment. These gases accumulate in the air and undergo a series of complex chemical reactions with the dust, small particles, bacteria, etc. in the air to form small agglomerated particles. When their concentration reaches a certain level and is further affected by weather, smog could appear. These hazardous gases include nitrogen dioxide (NO_2_), carbon monoxide (CO), nitric oxide (NO), carbon dioxide (CO_2_) and sulfur dioxide (SO_2_), etc. Some of these gases are irritating and toxic gases which are the main source of acid rain. Some have strong oxidizing properties and are strong combustion aids and some gases can cause greenhouse effects. They harm people’s health and pollute the environment, thus the adsorption and detection of these gases are of great significance to environmental protection and human health. From the perspective of materials, it is very important to find material substrates with high sensitivity to these gases. At present, nanoclusters, nanosheets, nanotubes and other low-dimensional nanomaterials are regarded to be potential candidates for these applications [1,2,3]. 

AlN is a kind of semiconducting material with excellent physical and chemical properties and its unique nanostructures have attracted the attentions of gas sensing researchers [4,5,6,7,8,9,10,11]. The electronic properties of nanomaterials can be improved by atomic modification or doping [12,13,14,15]. For example, Rezaei-Sameti et al. studied the adsorption characteristics of pure, B-, As-doped AlN nanotubes for CO adsorption [16]. The results showed that doping with B and As atoms was conducive to CO gas adsorption and effectively improved the sensitivity of AlN nanotubes to CO gas. Saedi et al. [17] considered the adsorption of H_2_S, COS, CS_2_ and SO_2_ gases by Al_12_N_12_ nanoclusters using density functional theory and it had been revealed that the Al_12_N_12_ nanocluster was a promising SO_2_ gas and electronic sensor and a CS_2_ gas electronic sensor, while they had different effects on conductivity. The theoretical investigations of AlN-based low-dimensional nanomaterials in this field are not only interesting to unveil some physics in the area but also of significance to move forward the field.

Based on previous research on AlN nanoclusters [18], in this work we have investigated the adsorption behaviors of NO_2_, NO, CO_2_, CO, and SO_2_ molecules on the large Al_60_N_60_ cluster. We select more stable adsorption sites to study their adsorption characteristics and electronic structures. Beside the pure cluster, some heteroatoms such as Ti, Si and Mn are also used to modify the Al_60_N_60_ nanocluster as substrates, for each of which the most stable sites are respectively considered to adsorb the above five gases and the sensitivity of each modified cluster to the gas molecules are also studied systematically.

## 2. Theoretical Methods 

In this work, the first-principles method based on Density Functional Theory (DFT) [19,20] is used. The Vienna ab-initio simulation package [21,22,23] is employed to optimize the geometry structures and calculate the energetics and electronic structures of various gas adsorption on pure and hetero-atom modified nanoclusters. The projector augmented wave (PAW) [21] pseudopotentials are used to describe the ion-electron interactions. The exchange correlation potentials are treated by the Perdew-Burke-Ernzerhof (PBE) functional within the framework of the generalized gradient approximation [24]. Due to the van der Waals interaction between the atomic cluster and the gas molecule, we have used DFT-D2 method of Grimme [25] to make the correction. The plan wave basis set with an energy cut-off of 520 eV has been used. The Brillouin zone is sampled using the Monkhorst-Pack method and the supercell approach is used for structural optimizations and electronic structure calculations. The conjugate gradient algorithm is used to optimize the geometry structures and the convergence criterion of the atomic force is less than 0.02 eV/Å. The charge transfer between Al_60_N_60_ nanocluster and hazardous gas molecules were analyzed by the Bader charge analysis using the previous method [26,27,28]. In order to avoid interaction between clusters in x, y and z directions, all the models are separated by the vacuum space of 20 Å. The following formula is utilized to calculate the adsorption energies:

E_ad_ = E_cluster+gas_ − E_cluster_ − E_gas_ (1)

where E_cluster+gas_ represents the total energy of the cluster (pure or hetero-atom modified) after gas molecule adsorption, E_cluster_ and E_gas_ represent the energies of the bare cluster and gas molecule respectively.

## 3. Results and Discussion

### 3.1. Adsorption of NO_2_, NO, CO_2_, CO and SO_2_ by Pure Al_60_N_60_ Nanocluster

**Figure 1:**
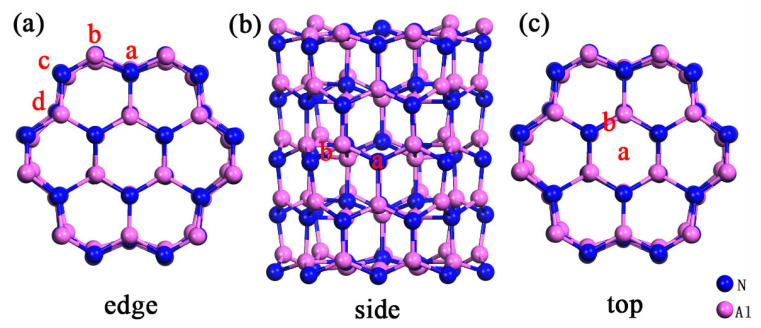
Various adsorption sites (shown in red letters) at each position (**a**) edge, (**b**) side or (**c**) top of pure Al_60_N_60_ cluster.

Firstly, we have systematically considered the adsorption of NO_2_, NO, CO_2_, CO and SO_2_ on the surface of pure Al_60_N_60_ nanoclusters, the structure of which had been revealed by us in an earlier study. There are different adsorption positions (we designate them as the edge, side and top position in this work) on the surface of clusters, and these three positions have four, two and two different adsorption sites, respectively. In Figure 1, Figure 1a shows the four adsorption sites at the edge position: edge.a (N-site) and edge.b (Al-site) have four coordination, edge.c (N-site) and edge.d (Al-site) have three coordination. Figure 1b shows the adsorption sites of the side position: side.a and side.b are the sites at the concave N atom and the Al-N bond, respectively. Figure 1c illustrates the two sites at the top position: top.a and top.b are the sites at the center and the Al-N bond, respectively. The NO_2_, NO, CO_2_, CO and SO_2_ molecules are horizontally or vertically placed in front of the edge and side sites and are horizontally placed directly above the top two sites for adsorption. Here, the readers may ask why we don’t adopt the cluster model with passivated heteroatoms or functional group such as H, O, OH, etc. in this study, since the cluster may react in air or solution. There are mainly two reasons: one is that the real cluster structure after passivation in air or solution would be very complicated, i.e., it is hard to define what kinds of heteroatoms or functional group (H, O, OH, etc.) passivate which specific sites of the cluster. It may be argued that this can be run through in every possible “combination”, but the computational time and cost would be huge considering that only for one model there will be 14 different adsorption configurations for each kind of gas molecule (more details will be in the following paragraph) and we have five different gas molecules to investigate in total. The other is that in our intrinsic cluster model, most surface Al/N atoms have three or four coordinations and the Al-N bonds are almost saturated, similar case also exists in other research [17]. Thus in this fundamental study we employ the current model to explore the intrinsic adsorption properties of Al_60_N_60_ nanocluster.

**Figure 2:**
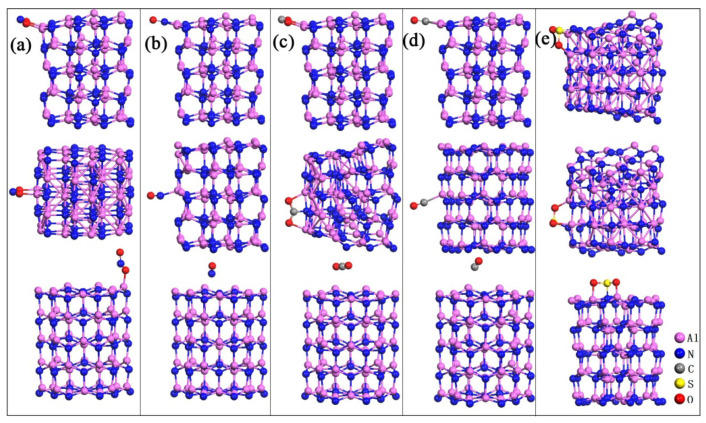
The configurations of pure Al_60_N_60_ clusters adsorbing hazardous gas molecules at three positions with higher adsorption energy values: (**a**) NO_2_, (**b**) NO, (**c**) CO_2_, (**d**) CO, (**e**) SO_2_. Top down in each subbox: the edge, side and top adsorption positions of clusters respectively.

Based on the above model, there are 14 different adsorption configurations for each kind of gas molecule. We have systematically studied the adsorption characteristics of each gas at various sites, as shown in Table 1. The subscripts h and p represent the horizontal and perpendicular adsorption of gas molecules at each site. From the data of adsorption energy, it can be seen that compared with other four gases, the adsorption energy absolute values of pure Al_60_N_60_ cluster towards CO are generally smaller. The adsorption energies of NO_2_, NO, CO_2_ and CO are relatively high at edge.a site. In addition, it is also found that when these five gas molecules are adsorbed at the top position, the adsorption energy value obtained at the Al-N bond site is high. When NO_2_ and CO_2_ are adsorbed at the side sites and SO_2_ is adsorbed at the edge.c_h_ and side.a_p_ sites, the adsorption energy values are more than 10 eV. Therefore, we have studied the adsorption details of these five gases at these sites. The optimized configurations corresponding to the higher energy values are shown in Figure 2. It can be seen from the Figure 2 that when the adsorption energy values are large, the corresponding optimized configurations have obvious shrinkage deformation such as NO_2_ adsorption at the side.a_h_ and side.b_h_ sites, CO_2_ adsorption at the side.a_p_ and side.b_h_ sites, and SO_2_ adsorption at the edge.c_h_ and side.a_p_. When NO_2_ is adsorbed at the edge.a_h_ site, the structure of the edge position is obviously deformed. From the optimized model structure, it can be seen that when the gas molecules are located at the edge and side sites, the structure is prone to deform.

In order to further study the electronic structures of the pure Al_60_N_60_ cluster adsorbing these five gases, we have selected the relatively stable structures when the Al_60_N_60_ cluster adsorbs those gas molecules. After screening, five configurations were obtained: NO_2_, NO, CO_2_, CO and SO_2_ adsorbed at the cluster top.b, edge.a_p_, edge.a_h_, edge.a_p_ and edge.a_h_ sites, respectively. Compared with other sites of the same gas, these structures are more stable and the adsorption energy values are higher. Based on this, the electronic properties are further studied.

**Figure 3:**
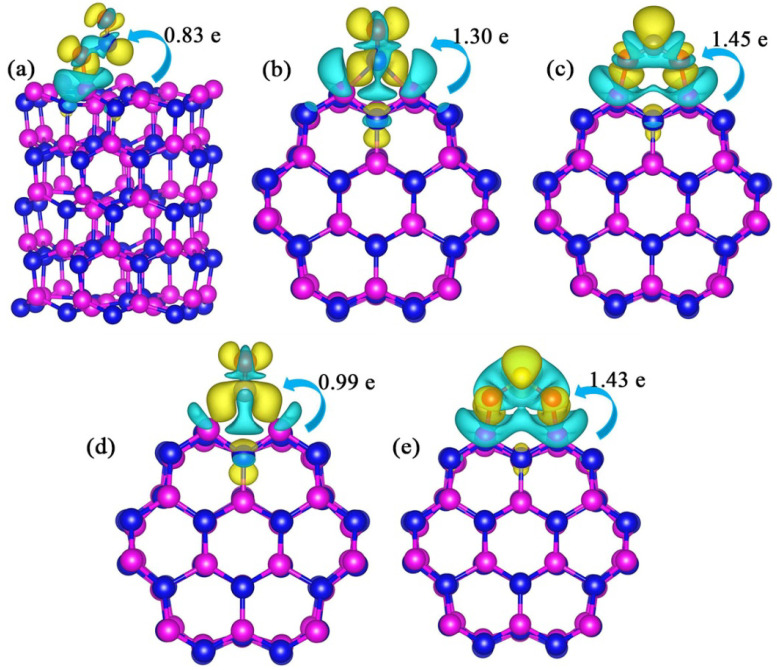
Differential charge density and charge transfer of (**a**) NO_2_ adsorbed at top.b site, (**b**) NO adsorbed at edge.a_p_ site, (**c**) CO_2_ adsorbed at edge.a_h_ site, (**d**) CO adsorbed at edge.a_p_ site and (**e**) SO_2_ adsorbed at edge.a_h_ site. Yellow and blue represent the charge accumulation and depletion respectively. The isosurface value is 0.0025 e/Å^3^. The arrow and charge number in the figure indicate the direction and value of the charge transfer between the nanocluster and the gas molecule.

**Figure 4:**
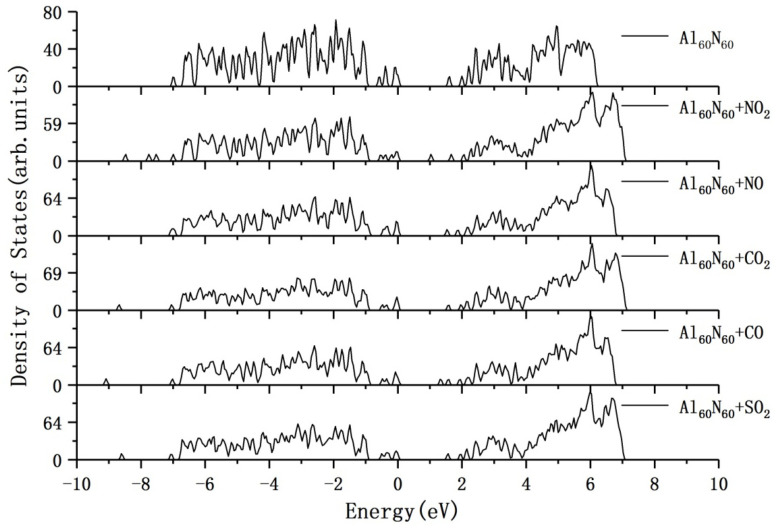
The electronic density of states of pure Al_60_N_60_ cluster before and after adsorption of various gas molecules. The Fermi level is set at zero.

In Figure 3, the differential charge density and charge transfer of the five gas molecules adsorbed at the relatively stable sites are illustrated. The O atoms of NO_2_, CO_2_ and SO_2_ are bonded with the Al atom at the edge of cluster, and the charge density of O atom after bonding is spindle-shaped. When the pure Al_60_N_60_ cluster adsorbs NO_2_, NO, CO_2_, CO and SO_2_, charge is transferred from the nanocluster to the gas molecules. The charge transfer of these five systems is not small. We also calculated the electronic structures of these five systems, as shown in Figure 4. After the pure Al_60_N_60_ cluster adsorbs NO_2_, the electronic structure changes significantly. From the density of states curve, it can be seen that after the adsorption of NO_2_, a new energy level appears between the Fermi level and the conduction band bottom. Before adsorption, the energy gap of pure Al_60_N_60_ cluster is 1.297 eV. After adsorption, the energy gap of the system becomes 0.721 eV and the change rate of gap is 44.40%. The pure Al_60_N_60_ cluster is relatively sensitive to NO_2_. When the pure Al_60_N_60_ cluster adsorbs CO_2_, NO and SO_2_, the energy gap change by 0.035 eV, 0.08 eV and 0.035 eV, respectively. Before and after the adsorption of CO by pure Al_60_N_60_, the energy gap changes by 0.945 eV and the change rate is 27.14%.

### 3.2. Atomic Modification of Al_60_N_60_ cluster by Ti, Si and Mn

**Figure 5:**
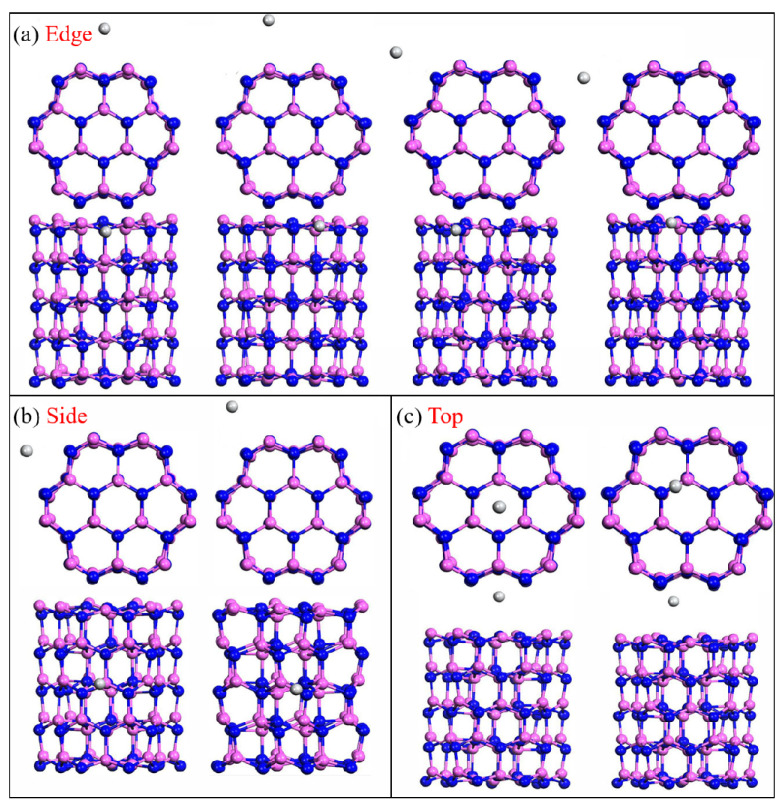
Top view and side view of the edge, side and top positions of Al_60_N_60_ cluster adsorbing single M atom (M atom is Ti, Si and Mn respectively): (**a**) Edge: From left to right, the M atom is located in front of the four-coordinated N atom, two-coordinated Al atom, three-coordinated N atom and three-coordinated Al atom at the edge position, respectively. (**b**) Side: From left to right, M atom is located in front of the Al atom recessed and Al-N bond at the side position, respectively. (**c**) Top: From left to right, M atom is located above the center of the top six-member ring and the top Al-N bond, respectively. The pink, blue and silver-white spheres represent Al, N, and M atoms, respectively.

**Figure 6:**
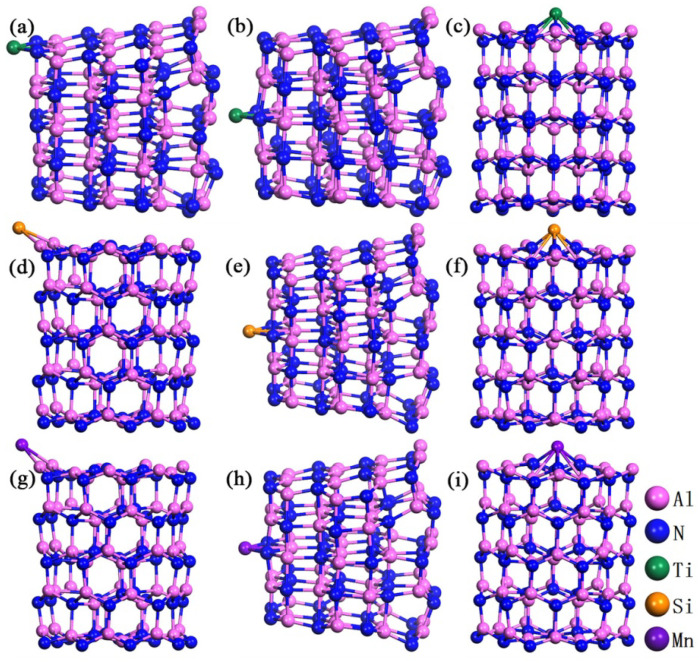
The optimized configurations of Al_60_N_60_ cluster adsorbing M atom at three positions with higher adsorption energy values: Ti atom is located at the (**a**) edge, (**b**) side, (**c**) top position of Al_60_N_60_ cluster. Si atom is located at the (**d**) edge, (**e**) side, (**f**) top of Al_60_N_60_ cluster. Mn atom is located at the (**g**) edge, (**h**) side, (**i**) top of Al_60_N_60_ cluster.

In order to further improve the adsorption and sensitivity of the cluster to those five gases, in this work we have tried to modify the Al_60_N_60_ cluster with Ti, Si and Mn heteroatoms and systematically calculated the adsorption energy, adsorption distance, bond length, bond angle and electronic structure of each system. Since the surface of the Al_60_N_60_ cluster has many different sites, first of all, we have to determine at which site each heteroatom is prone to occupy and which structure is relatively stable. These two aspects are discussed in the following research. When Ti, Si or Mn heteroatoms are adsorbed at the edge, side and top positions of Al_60_N_60_ cluster surface, there are four, two, and two adsorption sites, respectively, as shown in Figure 5. We have studied these eight adsorption structures and calculated the adsorption energy of each structure as shown in Table 2. It is found that when a Ti, Si or Mn atom is adsorbed at the side.a site the respective adsorption energies are –14.17 eV, –12.35 eV and –11.38 eV, which shows that the adsorption energy values of these three adsorption systems are generally higher. When these heteroatoms are adsorbed at some sites of the cluster, the adsorption energy is about 10 eV higher than other sites. To know the reason why the adsorption energy changes so much, we have studied the structures after adsorption and selected the geometrically optimized configurations with stable adsorption of the three hetero-atoms at the edge, side and top positions of the cluster as shown in Figure 6. When the cluster adsorbs Ti, Si or Mn atom at the side position, the structures shrink greatly. After the cluster edge.d site adsorbs a Ti atom, the structure also deforms obviously. The system with obvious deformation of structure after optimization has a relatively high adsorption energy value and unstable structure. Next, we will further study them from the aspect of electronic structures.

**Figure 7:**
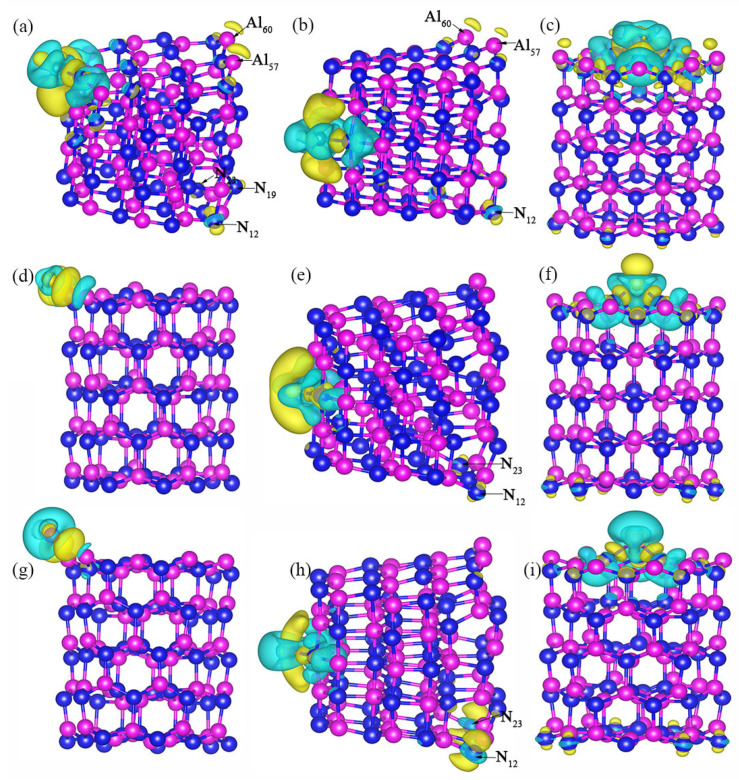
Differential charge density of Al_60_N_60_ cluster with higher adsorption energy values for single Ti, Si or Mn atom at three positions: (**a**) edge, (**b**) side, (**c**) top adsorption of Ti atom. (**d**) edge, (**e**) side, (**f**) top adsorption of Si atom. (**g**) edge, (**h**) side, (**i**) top adsorption Mn atom. Yellow and blue represent the charge accumulation and depletion (isosurface = 0.0025 e/Å^3^).

Figure 7 is the differential charge density diagram corresponding to the higher adsorption energy values when Ti, Si or Mn atom is adsorbed on an Al_60_N_60_ cluster. When three kinds of atoms are adsorbed at the side position of the cluster and Ti atom is adsorbed at the edge, a small amount of charge accumulation will appear at the No.60 and No.57 Al atoms at the far end, and charge depletion will occur at the No.12, No.19 and No.23 N atoms, and the charge distribution will be spindle-like. When a Ti, Si or Mn atom is adsorbed at the top of the cluster, the charge distribution is more uniform. There is a charge aggregation phenomenon between the three kinds of atoms and the Al_60_N_60_ cluster, and the Ti, Si and Mn atoms are bonded with the N atom at the top position of the cluster. Thus, when Ti, Si or Mn atom is adsorbed at the top of the Al_60_N_60_ cluster, they can have a relatively stable structure and the charge distribution is uniform. In the next section, we will select these configurations of hetero-atom modified at the top position of the Al_60_N_60_ cluster to carry out their adsorption properties towards hazardous gas molecules.

### 3.3. Investigation on the Adsorption of Hazardous Gas Molecules by Ti, Si or Mn-Modified Al_60_N_60_ Cluster

**Figure 8:**
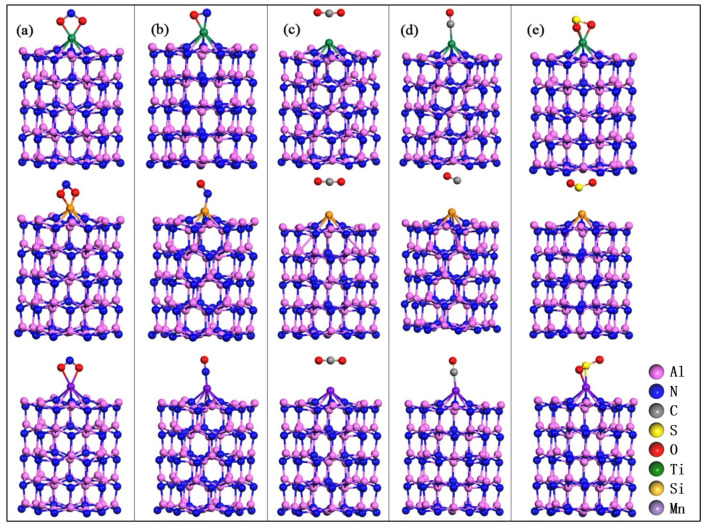
The optimal configurations of (**a**) NO_2_, (**b**) NO, (**c**) CO_2_, (**d**) CO and (**e**) SO_2_ adsorption on Al_60_N_60_ cluster modified by Ti, Si and Mn atoms. Top down: Ti, Si and Mn adsorption systems respectively.

NO_2_, NO, CO_2_, CO, and SO_2_ gas molecules are adsorbed at the stable sites of Al_60_N_60_ clusters modified by Ti, Si or Mn atom. After structural relaxations, the optimized configurations are shown in Figure 8. After relaxations, the heteroatom-modified Al_60_N_60_ clusters are not sensitive to CO_2_ and do not easily adsorb CO_2_; the Si-Al_60_N_60_ cluster does not easily adsorb CO and SO_2_, which can be further proved from Table 3. The M-Al_60_N_60_ cluster adsorbs CO_2_ with a smaller adsorption energy value, which is physical adsorption. Moreover, CO_2_ has a longer adsorption distance from the substrate. Compared with the structure before adsorption, CO_2_ is repelled by the substrate. The adsorption energy value of Si-Al_60_N_60_ cluster towards CO or SO_2_ is relatively small, and the adsorption distance is relatively long, which is weak adsorption. When the M-Al_60_N_60_ cluster adsorbs these five gas molecules, the bond length of the gas is elongated. Except the linear NO and CO, the bond angles of other molecules shrink. As can be seen from the table, the Ti-modified Al_60_N_60_ cluster has higher adsorption energy values when adsorbing NO_2_, NO, CO and SO_2_.

**Figure 9:**
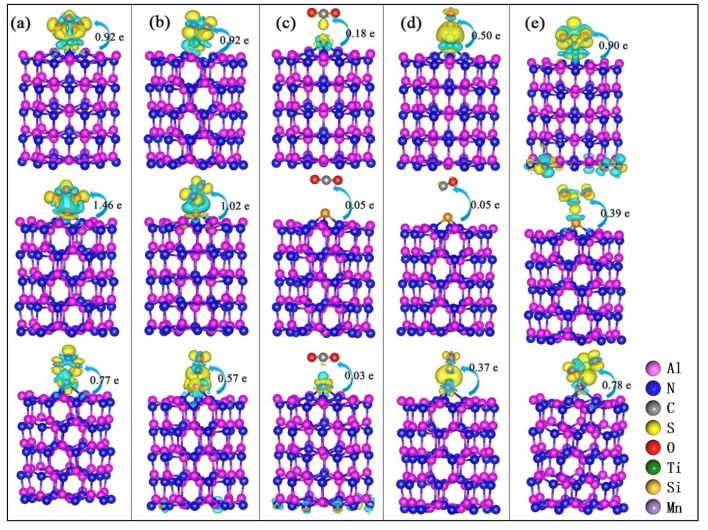
Differential charge density and charge transfer of (**a**) NO_2_, (**b**) NO, (**c**) CO_2_, (**d**) CO and (**e**) SO_2_ adsorption on the Al_60_N_60_ cluster modified by Ti, Si or Mn atom. Top down in each subbox: Ti, Si and Mn adsorption systems respectively. Yellow and blue represent the charge accumulation and depletion (isosurface = 0.0025 e/Å^3^). Arrows and values represent the direction and amount of charge transfer.

In order to further study the mechanism of M-Al_60_N_60_ clusters adsorption of those gas molecules, we have analyzed the differential charge density and charge transfer of M-Al_60_N_60_ clusters adsorbing NO_2_, NO, CO_2_, CO and SO_2_, respectively. As can be seen from Figure 9, the charge in all systems is transferred from the modified Al_60_N_60_ cluster to the gas molecule. The system of M-Al_60_N_60_ cluster adsorbing CO_2_ and Si-Al_60_N_60_ cluster adsorbing CO have smaller charge transfer and weaker interactions between gas and matrix. The system of Si-Al_60_N_60_ cluster adsorbing CO_2_ and CO hardly shows charge aggregation and loss. The Al_60_N_60_ clusters modified with Ti, Si or Mn atom do not easily adsorb CO_2_. When M-Al_60_N_60_ clusters adsorb NO_2_, NO and SO_2_, the charge transfers are large and there are strong interactions between gas and matrix. When Ti-Al_60_N_60_ and Mn-Al_60_N_60_ cluster adsorb those molecules, charge aggregation occurs between the gas and the matrix. When the Si-Al_60_N_60_ cluster adsorbs NO_2_ and NO, there is charge depletion between gas and Si-Al_60_N_60_ cluster. When M-Al_60_N_60_ clusters adsorb NO_2_ and SO_2_, the O atoms can form bonds with the heteroatom M. When the M-Al_60_N_60_ clusters adsorb NO, the N atoms easily form bonds with the M atom and the N atoms gain electrons.

**Figure 10:**
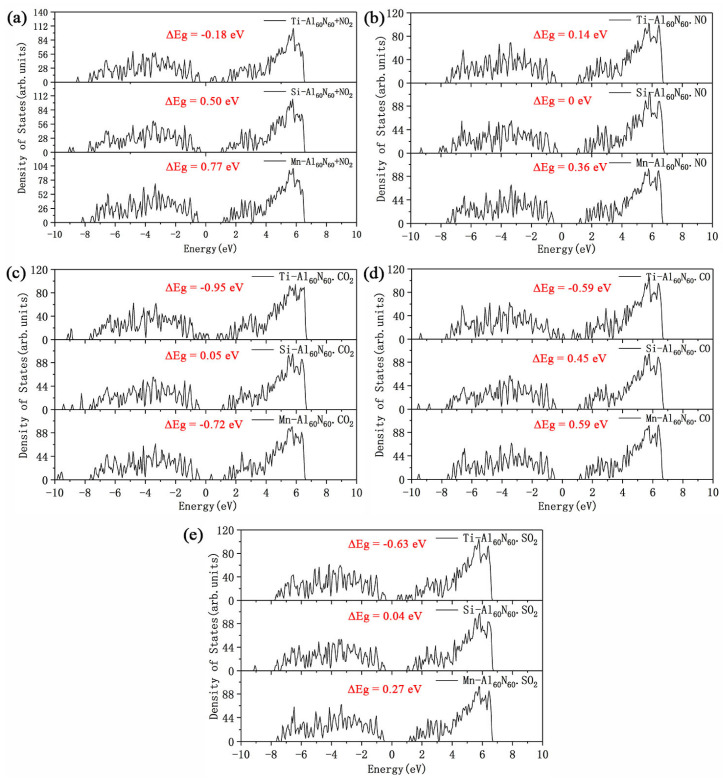
Density of states of various M-Al_60_N_60_ clusters adsorbing five gas molecules respectively: (**a**) NO_2_; (**b**) NO; (**c**) CO_2_; (**d**) CO; (**e**) SO_2_. The Fermi level is set at zero. ΔEg represents the energy gap change of the system before and after hetero-atom (M) modification.

To further figure out the electronic structure changes of the Ti, Si or Mn-modified Al_60_N_60_ clusters adsorbing those five hazardous gas molecules, we have calculated their corresponding density of states as shown in Figure 10. It can be seen from the figure that for the M-Al_60_N_60_ clusters adsorbing NO_2_, NO, CO_2_, CO and SO_2_, the energy gap of the system varies and the effects of three modifying atoms on the electronic structures are also different. In the figure, ΔE_g_ represents the energy gap change of the system before and after hetero-atom modification and the formula is as follows:

ΔE_g_ = E_g(M-Al_60_N_60_+gas)_ − E_g(Al_60_N_60_+gas)_ (2)

where E_g(M-Al_60_N_60_+gas)_ represents the energy gap of the gas adsorption system by modified Al_60_N_60_ cluster with M heteroatoms, E_g(Al_60_N_60_+gas)_ represents the gap of the system by pure Al_60_N_60_ cluster. It is found that for the Ti-Al_60_N_60_ cluster adsorbing CO_2_, CO, and SO_2_ and the Mn-Al_60_N_60_ cluster adsorbing CO_2_, the energy gap of these four systems are sharply reduced and the energy gap change rate are 75%, 62%, 50% and 57% respectively. The change rate of energy gap of Ti-Al_60_N_60_ cluster adsorbing NO_2_ was 25%. While, the energy gap of Si-modified Al_60_N_60_ cluster after adsorbing NO is unchanged. The energy gaps of the Ti-Al_60_N_60_ cluster adsorbing CO_2_, CO and SO_2_ and the Mn-Al_60_N_60_ cluster adsorbing CO_2_ are decreased, but the Al_60_N_60_ clusters modified by Ti or Mn atom do not easily adsorb CO_2_. Therefore, it is regarded that the Ti-atom modification can improve the sensitivity and sensing of Al_60_N_60_ cluster towards CO and SO_2_ and have potential to be used as a choice of related gas-sensing materials design.

## 4. Summary and Outlook

We have systematically investigated the atomic scale configurations and electronic structures of various hazardous gas molecules adsorbed by pure and heteroatom-modified Al_60_N_60_ clusters with Ti, Si and Mn using Density Functional Theory. It is found that for the pure Al_60_N_60_ cluster adsorbing those gas molecules, when NO_2_, NO, CO_2_, CO and SO_2_ are adsorbed on the top.b, edge.a_p_, edge.a_h_, edge.a_p_ and edge.a_h_ sites of the nanocluster, respectively, the corresponding structures are relatively stable and the charge transfer is relatively large. The chemisorption between Al_60_N_60_ cluster and gas molecule in these five systems is strong. The energy gap of pure Al_60_N_60_ cluster is reduced by 44% when adsorbing NO_2_ at its stable site, which is expected to be a candidate gas sensing material for NO_2_. When Ti, Si and Mn atoms are respectively adsorbed on the top sites of Al_60_N_60_ cluster, the corresponding structures are relatively stable and the charge distribution is uniform. When Ti-modified Al_60_N_60_ cluster adsorbs CO and SO_2_ gas molecules, the energy gap decreases sharply. Compared with the pure cluster adsorption system, the energy gap changes by 62% and 50% respectively, which greatly improves the sensitivity of Al_60_N_60_ nanocluster to CO and SO_2_. This theoretical work is proposed to supply fundamental clues and guide for experimentalists to develop appropriate nanoclusters for environmental and health applications.

## Figures and Tables

**Table 1:** The adsorption energies of various gas molecules adsorbed on different sites of pure Al_60_N_60_ nanocluster.

Sites														
E_ad_ (eV)	Edge								Side				Top	
	a_h_	a_p_	b_h_	b_p_	c_h_	c_p_	d_h_	d_p_	a_h_	a_p_	b_h_	b_p_	a	b
E_ad+NO_2__	−4.77	−4.41	−4.41	−3.39	−2.98	−2.88	−4.22	−3.17	−18.21	−0.22	−10.77	−2.79	−0.31	−3.32
E_ad+NO_	−2.02	−2.60	−2.01	−1.99	−2.01	−1.97	−1.58	−0.17	−0.81	−1.38	−0.83	−0.83	−0.36	−0.38
E_ad+CO_2__	−2.36	−0.12	−0.11	−0.09	−0.17	−0.28	−0.90	−0.37	−0.35	−10.04	−10.04	−0.25	−0.29	−0.30
E_ad+CO_	−0.99	−1.47	−0.97	−0.97	−0.09	−0.75	−0.08	−0.26	−0.74	−0.16	−0.75	−0.12	−0.13	−0.23
E_ad+SO_2__	−4.49	−3.95	−2.36	−1.00	−13.90	−1.78	−2.18	−1.79	−3.76	−10.82	−1.61	−3.23	−0.44	−3.43

**Table 2:** Adsorption energies of Ti, Si or Mn hetero-atom at different sites of Al_60_N_60_ cluster.

Sites								
E_ad_ (eV)	Edge				Side		Top	
	a	b	c	d	a	b	a	b
E_ad+Ti_	−3.21	−3.20	−3.36	−14.67	−14.17	−5.53	−4.80	−4.82
E_ad+Si_	−3.64	−3.63	−2.55	−2.82	−12.35	−6.63	−4.33	−3.40
E_ad+Mn_	−2.58	−2.59	−1.61	−1.94	−11.38	−3.02	−2.95	−2.67

**Table 3:** The adsorption energy (E_ad_), adsorption distance (d), bond length (d_(X-O)_), and bond angle (∠_(O-X-O)_), (X represents N, C or S atom). Adsorption distance is defined as the shortest distance from an atom of gas molecule to an atom in the modified matrix.

Systems	E_ad_ (eV)	d (Å)	d_(x-o)_ (Å)	∠_(O-X-O)_ (°)
Ti-Al_60_N_60_+NO_2_	−3.99	1.98	1.35	105.95
Si-Al_60_N_60_+NO_2_	−2.25	1.80	1.36	101.44
Mn-Al_60_N_60_+NO_2_	−2.89	2.17	1.28	111.62
Ti-Al_60_N_60_+NO	−3.37	1.88	1.37	180
Si-Al_60_N_60_+NO	−0.93	1.72	1.27	180
Mn-Al_60_N_60_+NO	−2.97	1.70	1.21	180
Ti-Al_60_N_60_+CO_2_	−0.12	2.98	1.19	172.22
Si-Al_60_N_60_+CO_2_	−0.15	3.35	1.18	177.44
Mn-Al_60_N_60_+CO_2_	−0.08	3.04	1.18	178.19
Ti-Al_60_N_60_+CO	−1.74	2.04	1.18	180
Si-Al_60_N_60_+CO	−0.07	3.35	1.15	180
Mn-Al_60_N_60_+CO	−1.44	1.90	1.17	180
Ti-Al_60_N_60_+SO_2_	−3.59	2.02	1.62	98.31
Si-Al_60_N_60_+SO_2_	−0.43	2.74	1.47	116.86
Mn-Al_60_N_60_+SO_2_	−2.06	1.93	1.54	113.89
